# Association of *Escherichia coli* pathotypes with fecal markers of enteropathy and nutritional status among underweight adults in Bangladesh

**DOI:** 10.3389/fcimb.2025.1553688

**Published:** 2025-04-10

**Authors:** Rahvia Alam Sthity, Md. Zahidul Islam, Md. Ehsanul Kabir Sagar, Md. Amran Gazi, Jafrin Ferdous, Md. Mamun Kabir, Mustafa Mahfuz, Tahmeed Ahmed, Ishita Mostafa

**Affiliations:** ^1^ Nutrition Research Division, International Centre for Diarrhoeal Disease Research, Bangladesh (icddr,b), Dhaka, Bangladesh; ^2^ Nutrition and Metabolism Graduate Program, University of Wisconsin-Madison, Madison, WI, United States; ^3^ Infectious Disease Division, International Centre for Diarrhoeal Disease Research, Bangladesh (icddr,b), Dhaka, Bangladesh; ^4^ Office of the Executive Director, International Centre for Diarrhoeal Disease Research, Bangladesh (icddr,b), Dhaka, Bangladesh; ^5^ Department of Public Health Nutrition, James P Grant School of Public Health, BRAC University, Dhaka, Bangladesh; ^6^ Faculty of Medicine and Health Technology, Tampere University, Tampere, Finland

**Keywords:** *Escherichia coli*, diarrhea, malnourished adults, environmental enteric dysfunction, Bangladesh

## Abstract

**Introduction:**

Environmental enteric dysfunction (EED), a subclinical intestinal disorder, is characterized by chronic fecal-oral exposure to entero-pathogens and could be diagnosed by measuring non-invasive biomarkers. *Escherichia coli* is the one of the key bacterial enteric pathogens that drives EED, but there is a lack of information on the *E. coli* pathotypes in relation to the biomarkers of EED in malnourished adults. Here, we intended to measure the possible association of these pathotypes with EED biomarkers and nutritional status of adults residing in a slum in Bangladesh.

**Method:**

Fecal samples were collected from 524 malnourished adults (BMI ≤18.5 kg/m^2^) living in a slum-setting in Dhaka from March 2016 to September 2019 and analyzed by TaqMan Array Card assays to evaluate the presence of E. coli pathotypes and other entero-pathogens. The multivariable linear regression model was used to assess the association.

**Results:**

In these malnourished adults, the most prevalent pathotype of E. coli was EAEC (61.7%) and the least prevalent was STEC (6.7%). The prevalence of atypical EPEC, ETEC and Shigella/EIEC were 52%, 48.9% and 45.1% respectively. The infection with atypical EPEC had significant positive association with levels of Myeloperoxidase (b = 0.38; 95% CI = 0.11, 0.65; p-value = 0.006). Similarly, a significantly higher concentration of alpha-1-antitrypsin (b = 0.13; 95% CI = 0.03, 0.22; p-value = 0.011) was found in the STEC-infected adults. However, no notable association was found between the *E. coli* pathotypes and nutritional status of these adult participants. Moreover, *Plesiomonas* infected adults were more likely to be infected with EAEC (p-value = 0.017), ETEC (p-value <0.001) and STEC (pvalue = 0.002). Significant coinfection was also detected among the pathotypes and other entero-pathogens such as *Giardia*, *Ascaris*, *Campylobacter*, *Salmonella*, *Enterocytozoon bieneusi*, and *Adenovirus*.

**Discussion:**

The study results imply that there is an influence of particular *E. coli* pathotypes (EPEC and STEC) on intestinal inflammation and gut permeability of the malnourished Bangladeshi adults, but no association with nutritional status is found. Potential pathogenicity of the E. coli pathotypes is also observed when co-infection with other pathogens exists in these adults.

## 1 Introduction

Diarrheagenic *Escherichia coli* (DECs) poses a significant public health threat to both children and adults, leading to the occurrence of diarrhea. These pathogens are most commonly transferred from human or animal waste to vulnerable hosts by contact with food and various environmental sources (such as water, soil, hands and flies) (Navab-Daneshmand et al., 2018). The disease mostly affects the developing world, particularly Africa, Asia, and Latin America. The 2015 Global Burden of Disease (GBD) study aimed to identify the causes of deaths associated to diarrheal disease in Bangladesh. It found that *E. coli* was one of the main factors responsible for these deaths in the region (Troeger et al., 2017). Child malnutrition, inadequate sanitation, contaminated water sources, poor water quality, and improper cooking methods, among other factors, contribute to the majority of cases (Croxen et al., 2013; Troeger et al., 2018). These enteric pathogens activate the immune system in the gut, resulting in both systemic and local inflammation. These conditions disrupt the normal functioning of the gut by increasing the permeability, decreasing the intestinal barrier function and damaging the structure of the gut. The term used to describe these conditions is Environmental Enteric Dysfunction (EED) (Budge et al., 2019).

One study found a strong correlation between increased levels of *E. coli* in drinking water and ready-to-eat meals and higher concentrations of fecal biomarkers of EED, as well as a higher EED composite score in children (Gizaw et al., 2022). To diagnose EED, the gold standard is an intestinal biopsy; however, it is an invasive method (Syed et al., 2016). Hence, a combination of biomarkers, such as Myeloperoxidase (MPO), Neopterin (NEO), Alpha-1 anti-trypsin (AAT), Calprotectin, and regenerative family member 1 beta (Reg1B), can be used to evaluate EED (Bartelt et al., 2019; Tickell et al., 2019). MPO, a neutrophil-specific marker, reacts with hydrogen peroxide to release highly cytotoxic substances that aid in the elimination of foreign microbes. Nevertheless, these harmful products can also harm healthy tissue and lead to inflammation in the gastrointestinal tract (Hansberry et al., 2017). The selection of fecal NEO as a marker for intestinal cell-mediated inflammation is based on its release from phagocytic cells by an IFN-dependent manner under circumstances involving cell-mediated inflammation (Seki et al., 1996). The concentration of AAT in the gut rises when there is an increase in permeability or when the mucosal barrier of the gut is damaged. Extravasation of serum into the gut and subsequent presence in fecal matter make it a suitable biomarker for assessing intestinal permeability (Wang et al., 2015). Calprotectin is a protein present in white blood cells that becomes active in the presence of inflammation. This characteristic makes calprotectin an efficient biomarker for identifying inflammation in the gut (Kapel et al., 2010; Jukic et al., 2021). Reg1B functions as an indicator, signaling damage to the epithelial tissue and subsequent repair mechanisms in the small intestine (Syed et al., 2016).

Although EED is typically acquired in early childhood, it can also continue into adulthood, and it was initially identified in the adults (Crane et al., 2015). In addition, underweight, a persistent issue in many developing nations, stems from various factors such as diarrhea, inadequate sanitation, and starvation (Black et al., 2013). DEC pathotypes are a primary contributor to diarrhea in adults, especially in developing countries (Nataro and Kaper, 1998). DEC strains have been classified into separate groups according to their distinctive virulence factors and phenotypic features. These categories comprise Enteroaggregative *E. coli* (EAEC), Enteropathogenic *E. coli* (EPEC), Enterotoxigenic *E. coli* (ETEC), Enteroinvasive *E. coli* (EIEC) and shiga-like toxin (STEC) (Gomes et al., 2016). PCR tests now easily identify the specific genetic features that define these pathotypes. EPEC is primarily associated with childhood diarrhea, while ETEC and EAEC are commonly associated with traveler’s diarrhea. EIEC is typically associated with a more severe disease, often characterized by bloody diarrhea. STEC is also associated with bloody diarrhea and is frequently accompanied by thrombotic thrombocytopenic purpura (TTP) and hemolytic uremic syndrome (HUS). Additionally, EAEC has been associated with persistent diarrhea and gastrointestinal disorders in developing countries (Croxen et al., 2013). EPEC consists of two different groups of organisms: typical EPEC (tEPEC) and atypical EPEC (aEPEC). The differentiation between tEPEC and aEPEC strains relies on the presence of the bundle-forming pilus (BFP), an adhesive structure essential for the adherence of bacterial strains to the epithelial cells of the intestine. Strains that exhibit BFP are categorized as tEPEC, whereas those that do not possess BFP are classified as aEPEC. aEPEC exhibits greater heterogeneity compared to tEPEC regarding phenotypic traits and virulence factors (Gomes and Pedrajo, 2012). tEPEC is more frequently isolated in diarrheal patients from developing countries, while aEPEC isolation is more prevalent in developed countries (Trabulsi et al., 2002; Kotloff et al., 2013). Prior studies conducted in different countries have examined the various DEC strains and their correlation with EED biomarkers. Researchers had previously reported an elevated plasma neopterin level in young Norwegians infected with ETEC (Rim et al., 2024).

Modgil et al. observed a notable rise in calprotectin levels in EAEC-infected mice isolated from symptomatic cases (Modgil et al., 2022). Gazi et al. conducted an analysis of various fecal markers of inflammation in children between the ages of 12 and 18 months. It was found that infection with specific strains of *E. coli* bacteria (EAEC and ETEC) had an impact on the gastrointestinal health of undernourished children in Bangladesh. ETEC infections resulted in elevated calprotectin levels and decreased NEO levels, whereas MPO levels appeared to remain unchanged (Gazi et al., 2022). The majority of existing studies have primarily examined the infection in newborns or children under five years of age, with less research available on adults. In addition, previous research has investigated potential correlations between specific *E. coli* pathotypes and EED biomarkers. However, to the best of our knowledge, no investigations have included all pathotypes in adults at the same time. Hence, our study aims to investigate the correlation between infections caused by different strains of *E. coli* and biomarkers indicating EED and nutritional status (body mass index, BMI) in undernourished adults residing in Bangladesh.

## 2 Materials and methods

### 2.1 Study design, site and population

The data used in this study was obtained from the Bangladesh Environmental Enteric Dysfunction (BEED) project, which was carried out in a slum neighborhood in Dhaka, Bangladesh. The BEED project was a community-based nutrition intervention trial that included adults with malnutrition (BMI ≤18.5 kg/m^2^). The study spanned from March 2016 to September 2019, and the participants’ ages ranged from 18 to 45 years. Fecal samples were collected from 524 participants for this investigation. The study excluded individuals with severe anemia, tuberculosis, psychiatric disorder, and any chronic conditions. The study excluded pregnant and lactating women, as well as individuals diagnosed with cancer. During the enrollment process, our trained field workers collected socio-demographic information and anthropometric data. The BEED study aims to assess the effectiveness of nutrition intervention in enhancing the nutritional status of the participants, exploring the contribution of enteric pathogens to the development of EED and malnutrition, and creating a histological scoring system to detect EED and validate the score using non-invasive biomarkers of EED (Fahim et al., 2021). The detailed methodology of the BEED study was previously published (Mahfuz et al., 2017).

### 2.2 Laboratory assays

The laboratory experiments were conducted at the “Emerging Infections & Parasitology Laboratory” of icddr,b. Plasma was separated from blood samples using centrifuges at 4000 rotations per minute for 10 minutes. Plasma and stool samples were frozen at -80°C until analysis. Alpha-1-acid glycoprotein (AGP) (Alpco, Salem, NH), C-reactive protein (CRP) (Immundiagnostik, Bensheim, Germany), and ferritin (ORGENTEC Diagnostika GmbH, 55129 Mainz, Germany) were among the plasma biomarkers analyzed using ELISA. The plasma zinc concentration was quantified by atomic absorption spectrometry. Commercial ELISA kits were used to assess the fecal biomarkers for instance, MPO (Alpco, Salem, New Hampshire), AAT (Biovendo Chandler, North Carolina), NEO (GenWay Biotech, San Diego, California), Calprotectin (BUHLMANN fCAL, Schonenbuch, Switzerland) and Reg1B (TechLab, Blacksburg, Virginia). A quantitative PCR test employing TaqMan Array Cards (TAC) by Applied Biosystems (Life Technologies Corporation, Carlsbad, CA) was used to detect the presence of *E. coli* pathotypes and other enteropathogens in stool samples. This test is a 384-well singleplex probe-based real-time PCR. In this experiment, nucleic acid extraction was performed using a modified procedure that involved bead beating with the QIAamp Fast DNA Stool Mini Kit (Qiagen, Hilden, Germany) (Ijarotimi, 2013). Phocine herpes virus and MS2 bacteriophage were included as external controls during nucleic acid extraction to assess the efficacy of both the extraction and amplification processes. Contamination was assessed by monitoring extraction blanks and no-template controls. The cutoff for finding pathogens in the TAC test was a Ct-value of ≤35 which was seen as an indication of pathogen positivity. The previous literature has provided a detailed description of the performance and interlaboratory reproducibility of the TAC technique and the specifics on TAC design, including the primer sequences (Liu et al., 2013; Platts-Mills et al., 2017).

### 2.3 Variables used in this analysis

The main aim of this study was to assess the association of nutritional status (BMI) of Bangladeshi underweight adults and EED biomarkers with the presence of *E. coli* pathotypes in the feces. Accordingly, *E. coli* pathotypes were exposure variables and on the basis of the presence of *E. coli* pathotypes derived from TAC findings. In this study, our main outcome variables were the nutritional status of Bangladeshi adults (BMI) and fecal biomarkers (NEO, MPO, AAT, Reg1B and Calprotectin). For the nutritional status BMI model, age, sex, household monthly income, wash indices, EED biomarkers, zinc, and ferritin were considered covariates. On the other hand, for the EED biomarker model, the age, sex, household monthly income, wash indices (a score that was determined by answering five hygiene-related queries, e.g. handwashing, use of toilet paper etc.), inflammatory biomarkers (AGP, CRP), zinc, and ferritin were considered covariates.

### 2.4 Statistical analyses

The socioeconomic and demographic features were represented using frequency and percentage for categorical data, median with inter-quartile ranges (IQR) for asymmetric continuous data, and mean with standard deviation for symmetric continuous variables. The EED biomarkers were compared across the groups of *E. coli* pathotypes that tested positive and negative using either a t-test or a Mann-Whitney U test. The concentrations of fecal biomarkers (Reg1B, AAT, MPO, NEO and Calprotectin) were log transformed and made symmetric before being incorporated into the multivariate model. We applied multivariable linear regression to analyze the association between *E. coli* pathotypes and concentrations of EED biomarkers, as well as the relationship between *E. coli* pathotypes and the nutritional status indicator BMI in adults. All the models were adjusted by some covariates; covariates were selected based on available literature and their biological and clinical significance. A probability of 0.05 was considered to have statistical significance. The statistical studies were conducted using the STATA program, specifically version 15.0.

## 3 Results

A total of 524 patients were included in the specific analyses conducted as part of the EDD study in Bangladesh (Modgil et al., 2022). Out of all the participants, the proportion of female participants was the highest at 72%. The average age of the participants was 23.8 years with a standard deviation of 6.9 years (**Table 1**).

**Table 1:** Baseline characteristics of the malnourished adults.

Variables, n (%)	Participants (n=524)
Age in years, mean (SD)	23.8 (6.9)
Sex	
Female	381 (72.7%)
Body mass index, mean (SD)	17.3 (0.8)
Nutritional status	
Mild underweight	333 (63.5%)
Moderate underweight	167 (31.9%)
Severe underweight	24 (4.6%)
Monthly income in BDT, mean (SD)	16099.0 (9503.2)
Water treatment (Yes)	320 (61.1%)
Improved sanitation (Yes)	79 (15.1%)
Use of toilet paper (Yes)	115 (22.0%)
Always wash hand after toilet (Yes)	410 (78.2%)
Always wash hand before cooking or preparing foods (Yes)	88 (16.8%)
Crowding index	
Low (≤ 4 people sleep in a room)	437 (83.4%)
High (*>* 4 people sleep in a room)	87 (16.6%)
Separate space for kitchen (Yes)	458 (87.4%)
Kept chickens/ducks at home (Yes)	38 (7.3%)

SD, Standard Deviation.

### 3.1 Prevalence of *E. coli* pathotypes

**Figure 1:**
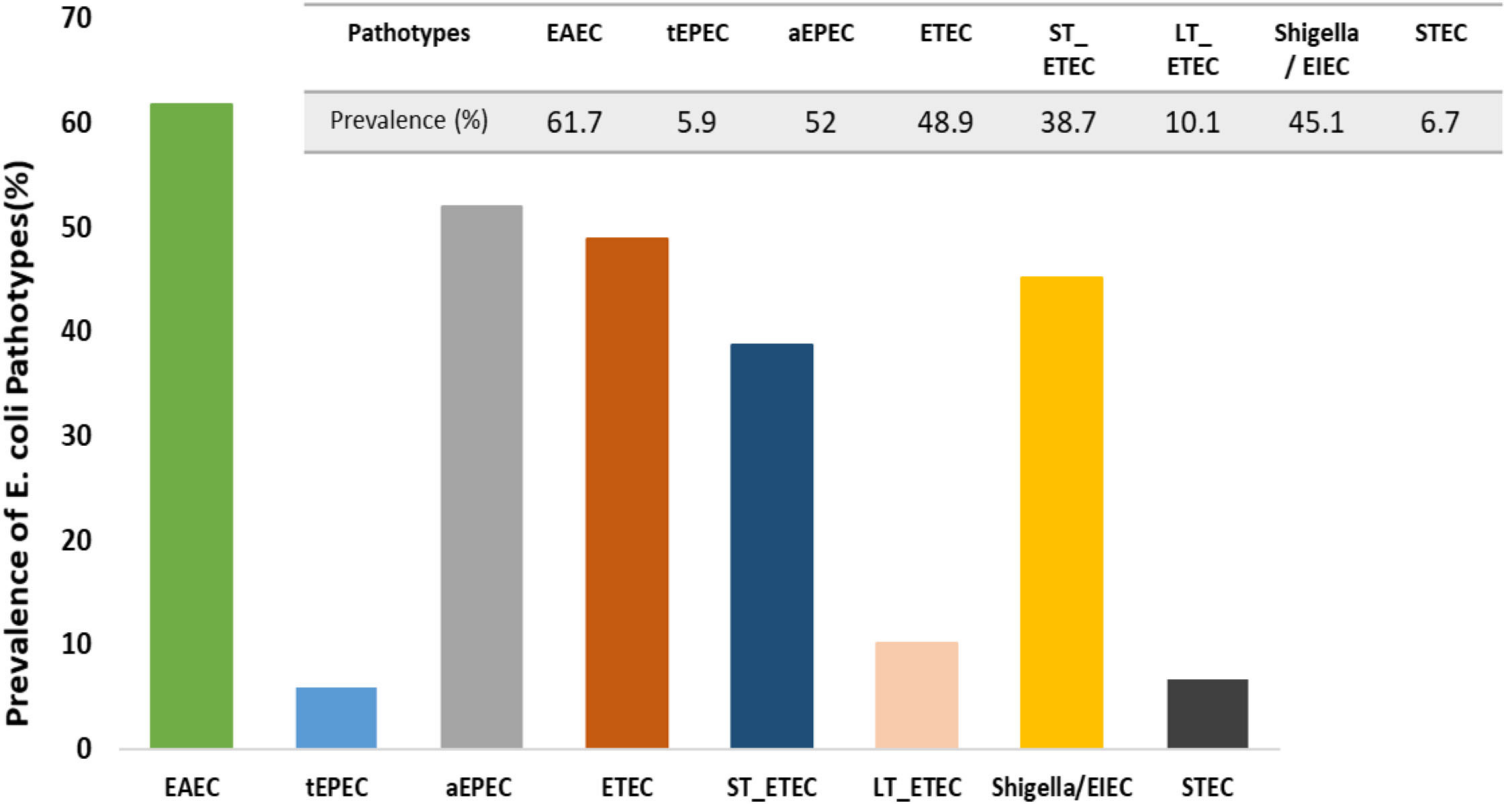
Prevalence of *E. coli* pathotypes among the participants.

Out of the many *E. coli* pathotypes, including EAEC, tEPEC, aEPEC, ETEC, ST_ETEC, LT_ETEC, *Shigella*/EIEC, and STEC, EAEC had the highest prevalence, accounting for 61.7% (**Figure 1**). The incidence of heat-stable ETEC (ST-ETEC) was greater at 38.7% compared to heat-labile ETEC (LT-ETEC) at 10.1%. In comparison, the prevalence of aEPEC was greater at 52%, whereas the prevalence of tEPEC was only 5.9%.

### 3.2 Coinfection among *E. coli* pathotypes and other enteropathogens

To investigate the coinfection of *E. coli* pathotypes with other enteric pathogens an analysis was conducted. Coinfections may affect disease severity, immunological responses, and intestinal inflammation, thereby altering the biomarker profiles identified in this investigation. The findings (**Supplementary Table S1**) indicate that EAEC was significantly coinfected with aEPEC (p = <0.001), ETEC (p = <0.001), and ST_ETEC (p = <0.001). Additionally, ETEC showed coinfection with aEPEC (p = 0.027), and aEPEC was found to be coinfected with ST_ETEC (p = 0.001). *Blastocystis* showed a considerable co-infection with aEPEC (p=0.001) and ST_ETEC (p=0.043), while *Plesiomonas* also exhibited significant co-infection with EAEC (p=0.014), ETEC (p=<0.001), and ST_ETEC (p=<0.001). Furthermore, there was a significant association between *Campylobacter*-Pan Genome and LT_ETEC (p = 0.024) as well as *Shigella*/EIEC (p = 0.016).

### 3.3 Distribution of fecal biomarkers among adults infected with *E. coli* pathotypes

This investigation revealed that EED biomarker levels exhibited no significant differences between Bangladeshi adults infected with EAEC or tEPEC and those without such infections (**Supplementary Table S2**, Graph SG1 And Graph SG2), indicating that these pathogens may not substantially affect EED responses as assessed by MPO, NEO, AAT, Calprotectin, and REG1B in this population. However, MPO concentrations were significantly higher in aEPEC-positive samples relative to negative samples (p = 0.027). Considering that MPO serves as a biomarker for intestinal inflammation, this observation implies a potential role for aEPEC in triggering an immunological response. Similarly, the notable rise in Calprotectin levels in Shigella/EIEC-positive cases (p = 0.007) indicates that Shigella/EIEC infections can lead to intestinal inflammation. The increased AAT levels in STEC-positive samples (p = 0.006) may suggest a correlation between STEC infection and intestinal permeability. No additional pathotypes of *E. coli* shown a significant correlation with any other biomarkers of EED.

### 3.4 Association of infection with *E. coli* pathotypes with fecal EED biomarkers and BMI

**Figure 2:**
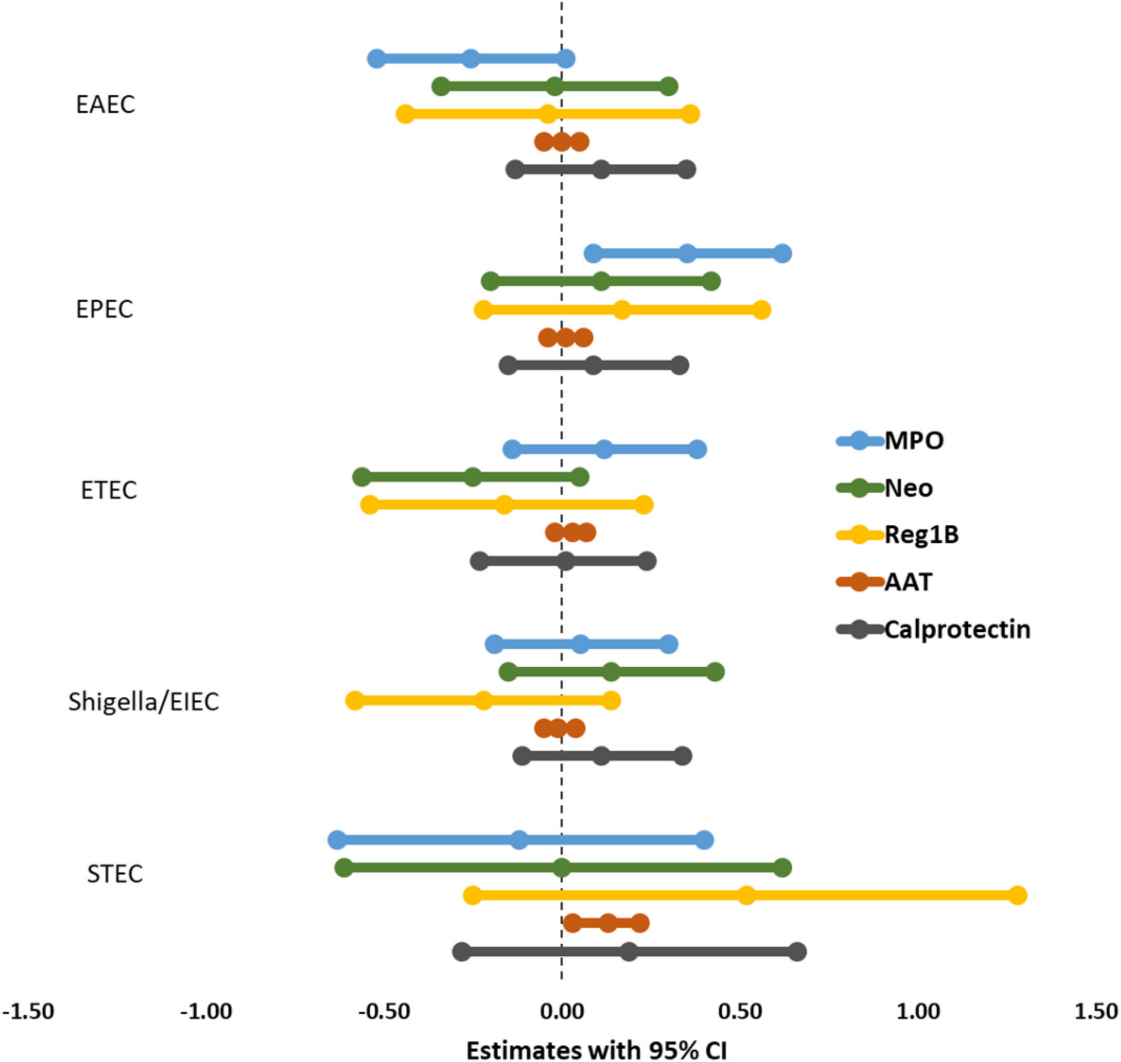
Association between *E. coli* pathotypes and fecal biomarkers of EED in malnourished adults.

Multivariate linear regression analysis, adjusting for covariates and potential confounders, demonstrated a significant positive correlation between EPEC infection and the fecal biomarker MPO (β = 0.35; 95 percent CI = 0.09, 0.62; p-value = 0.008), indicating that EPEC may play a role in neutrophil activation and intestinal inflammation (**Figure 2**; **Supplementary Table S3**). Likewise, STEC infection had a significant association with increased levels of the fecal biomarker AAT (β = 0.15; 95 percent CI = 0.05, 0.24; p-value = 0.003), supporting its function in intestinal permeability. No additional infections were found to be associated with the remaining biomarkers.

**Figure 3:**
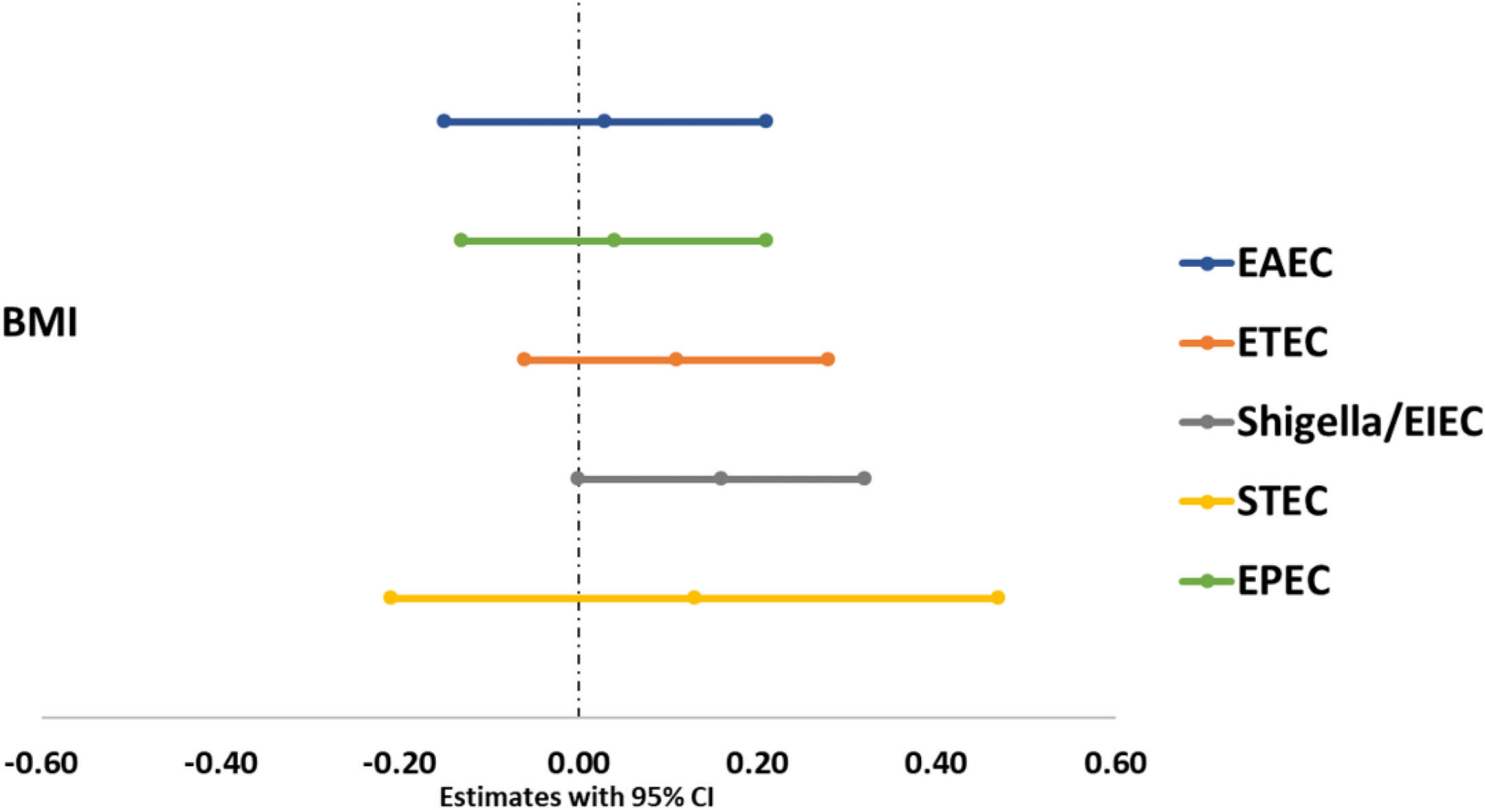
Association between *E. coli* pathotypes and nutritional status of adults.

Through a multivariate linear regression analysis that adjusted for covariates and potential confounders, we observed no significant association with BMI and *E. coli*, suggesting that *E. coli* pathotypes may not have a direct impact on BMI of this population (**Supplementary Table S4**; **Figure 3**)

The analysis of *E. coli* subtypes showed a notable positive correlation between aEPEC and the fecal biomarker MPO (β = 0.38; 95% CI = 0.11, 0.65; p = 0.006) (**Supplementary Table S5**). This finding indicates that aEPEC might trigger an intensified neutrophilic inflammatory response in the gastrointestinal tract, as MPO serves as a marker of neutrophil activity. Nonetheless, no other *E. coli* subtypes or additional EED biomarkers exhibited significant associations, suggesting possible variability in the inflammatory effects of different strains.

An alternative analysis was conducted using two subgroups: *E. coli* positive and negative (**Supplementary Tables S6**, **S7**), and the results were very similar to those in **Supplementary Table S2**. *E. coli* was defined as positive if any of the following were positive: EAEC, tEPEC, aEPEC, ETEC, ST_ETEC, LT_ETEC, *Shigella*/EIEC or STEC. The alternative was *E. coli* negative.

A multivariable regression analysis was performed separately for male and female participants to investigate potential variations in *E. coli* infections. However, the results were nearly identical (**Supplementary Table S8**).

## 4 Discussion

While there are several publications on diarrheal disease in children under the age of five, this study stands out as one of the few that examines the presence of five distinct DEC pathotypes in adults. Diarrhea may be a clinical symptom of EED, indicating exposure to enteric pathogens. However, this exposure can occur with or without diarrhea (Budge et al., 2019). Prior to this investigation, little to nothing was known about the correlation between DEC strains and EED markers in Bangladeshi adults.

This study found that infection with specific pathotypes of *E. coli* (EPEC, *Shigella*/EIEC, and STEC) affected gut health biomarkers in adults from Bangladesh. However, there is no correlation between these infections and underweight individuals living in a slum. The findings of our study indicate that the highest prevalence of infection was observed in adults infected with EAEC (61.71%), followed by EPEC (52%), ETEC (48.85%), *Shigella*/EIEC (45%), and STEC (6.67%). Prior research has indicated that EAEC and EPEC are significant contributors to acute intestinal infections in people residing in industrialized countries (Makarova, 2023). In Chhatak and Mathabaria, Bangladesh, a study found that children under five years and adults aged 20 to 60 years were most susceptible to ETEC diarrhea (Chakraborty et al., 2024). EAEC, EPEC, and ETEC were the most commonly identified pathogens in children having EED, according to a recent study conducted in a slum in Dhaka, Bangladesh (Gazi et al., 2022). Moreover, the present analysis demonstrates a greater occurrence of aEPEC in comparison to tEPEC. Recent research indicates that aEPEC is more common than tEPEC in cases of chronic diarrhea, regardless of whether it occurs in developing or developed nations (Ochoa et al., 2008). Besides, this study reveals that the prevalence of ETEC that expresses the heat-stable enterotoxin was high.

The higher occurrence of *E. coli* pathotypes identified in our study may be linked to co-infection among *E. coli* pathotypes or with other intestinal pathogens. *Shigella*/EIEC showed a strong association with *Salmonella*, *Campylobacter*-Pan Genome, *Enterocytozoon bieneusi*, *Trichuris*, *Adenovirus*-Pan Genome, and *Aeromonas*. Conversely, *Plesiomonas* exhibited strong correlations with EAEC, ETEC, and STEC. The *Campylobacter*-Pan Genome showed a high degree of co-infection with ETEC. The presence of *Ascaris* was found to have a substantial association with EPEC, while the presence of *Entamoeba histolytica* was found to have a significant association with ETEC. Similarly, *Blastocystis* showed a significant co-infection with both EPEC and ETEC, whereas *Isospora* showed a significant co-infection with EPEC and STEC. Additionally, there were cases of coinfections among *E. coli* pathotypes. Numerous diarrheal studies conducted worldwide have recognized dual infections with intestinal pathogens (Zhang et al., 2016; Ledwaba et al., 2018; Pijnacker et al., 2019). Concurrent infections not only worsen the progression of diseases but also impact the host’s immunological responses to pathogens, undermining the efficiency of disease control programs at the state or country level (Cox, 2001; Read and Taylor, 2001; Li and Zhou, 2013). In any species, the presence of many pathogens can result in more severe diarrhea compared to an infection caused by a single pathogen alone (Grimprel et al., 2008). Nyholm et al. discovered that Diarrheagenic *E. coli* has the ability to obtain virulence genes from other pathogroups by horizontal gene transfer (Nyholm et al., 2015). This process leads to the development of intermediate pathogroups (Müller et al., 2007), also known as hybrid (Mellmann et al., 2011). A study conducted in Pakistani children under the age of five identified co-infection of EPEC with EHEC and EAEC (Zil-e-Huma et al., 2019). A study conducted in children from rural and peri-urban populations in South Africa revealed the co-infection of DECs with *Campylobacter*, *Salmonella*, *Cryptosporidium parvum*, *Rotavirus*, and *Adenovirus* (Potgieter et al., 2023). The presence of EAEC has been detected in individuals who are infected with the human immunodeficiency virus (HIV), particularly in children and travelers in developing nations (Zil-e-Huma et al., 2019).

This study found a strong and positive correlation between the amount of MPO in feces and the presence of atypical EPEC. EPEC infections are commonly linked to inflammatory enteropathy and/or diarrhea in populations with limited resources (Platts-Mills et al., 2015; Rogawski et al., 2018). The diarrhea caused by EPEC infection is a result of elevated ion secretion, increased permeability of the intestines, inflammation in the intestines, and loss of absorptive surface area due to microvillus effacement (Kaper et al., 2004). Both neutrophils and monocytes may become activated by lipopolysaccharides produced by bacteria (Kothari et al., 2011). Researchers suggest that during phagocytosis MPO regulates the respiratory activity of these polymorphonuclear leukocytes (PMNs) (Butterfield et al., 1998). MPO is considered a significant mechanism for O_2_-dependent microbicidal action. The activation of PMNs leads to an abrupt rise in oxygen consumption, along with the production of reactive oxygen species (ROS) and the secretion of enzymes including elastase and MPO (Woods et al., 2003). Multiple prior studies have demonstrated that solubilized MPO from inflammatory tissue correlates directly with the quantity of neutrophils present (Weissmann et al., 1980; Krawisz et al., 1984). Consequently, an elevated amount of MPO in stool samples indicates intestinal inflammation (Arndt et al., 2016). An earlier study confirmed that elevated levels of fecal MPO correlate with an increased prevalence of enteric pathogens in stool, and this biomarker has been utilized as an indicator of EED (George et al., 2018). Our current finding is consistent with a prior murine model, where MPO levels were elevated during the acute phase of EPEC infection (Ledwaba et al., 2020). LT-ETEC and MPO levels exhibited a significant association in the MAL-ED birth cohort studies (Khalil et al., 2021).

The findings also demonstrated a noteworthy positive correlation between AAT and STEC. The pathogenic processes of STEC are associated with both adherence and toxin generation. STEC attaches to gastrointestinal epithelial cells using the outer membrane protein intimin (Karmali et al., 2010), which is similar to the adherence mechanisms observed in EPEC. Subsequently, the STEC toxin is transported to the kidney either through the bloodstream or by the movement of neutrophils (Hurley et al., 2001). Neutrophils, during the initial stage of diarrhea-associated hemolytic uremic syndrome, are stimulated and become more adhesive than usual. They affect the endothelium by generating elastase complexed with AAT (Zoja et al., 2010). Multiple studies indicate that *E. coli* can compromise the intestinal barrier and increase permeability within the gastrointestinal tract (Spitz et al., 1995; Mangell et al., 2002). Moreover, Alpha-1-antitrypsin functions as a protease inhibitor, exhibiting increased levels during mucosal barrier disruption and enhanced gut permeability (Barekatain et al., 2020; O’Brien et al., 2022). This could explain the reason why participants who tested positive for STEC had elevated levels of AAT. Increased fecal AAT levels are also observed in cases of diarrhea caused by enterotoxigenic *E. coli*, *Shigella*, *Salmonella*, *Rotavirus* and *Adenovirus*. These pathogens damage the tight junctions in the gastrointestinal tract, resulting in the leakage of proteins (Aulia et al., 2009; Rahmat et al., 2023). Furthermore, *Shigella*/EIEC-positive participants showed a notable rise in Calprotectin levels. EIEC exhibits numerous similarities to *Shigella*, particularly in terms of their virulence mechanisms (Okeke, 2009). However, the multivariable model could not establish a significant association between Calprotectin and *E. coli* pathotypes. Elevated fecal calprotectin levels in samples with various enteropathogens were observed in a study involving participants aged between three months and four years conducted in Southern India (Praharaj et al., 2018). Additionally, the presence of ETEC in stool was associated with increased fecal calprotectin concentrations in children aged 6–30 months in rural Bangladesh (George et al., 2018).

This study observed no statistically significant association between *E. coli* pathotypes and the nutritional status (BMI) of the individuals. A recent study on Bangladeshi children aged 12 to 18 months revealed a notable adverse correlation between EPEC and linear growth, as indicated by the length-for-age z score (LAZ), as well as underweight, as indicated by the weight-for-age z score (WAZ) (Trabulsi et al., 2002). The MAL-ED study discovered that EAEC exhibited a negative association with linear growth at 24 months of age (Das et al., 2021).

This study has several limitations. Initially, our research solely included persons who had a low BMI. The lack of a group of healthy controls reduces the robustness of the results we obtained. Consequently, we conducted an exploratory study using two subgroups (*E. coli*-positive and *E. coli*-negative), and the findings demonstrated consistency with the bivariate analysis (**Supplementary Table S2**). In addition, there is a significant disparity in the ratio of female to male participation. This community-based intervention trial involved male participants who were required to visit the study site every day in the morning for two months to receive the nutritional intervention. The majority of eligible males were those who earned wages for their families and declined to miss work for an hour, leading to an increased proportion of female participants, mainly homemakers or part-time workers. Moreover, the BEED study only enrolled undernourished adults who were not experiencing any acute illness at the time of enrollment. Therefore, all participants were asymptomatic, which limits our ability to directly assess the relationship between *E. coli* pathotypes and clinical symptoms. Future studies that include both symptomatic and asymptomatic individuals could provide further insights into high-risk groups and the potential role of biomarkers in disease progression.

This research’s strength is the use of quantitative TAC PCR assays to identify *E. coli* pathotypes and a large array of other intestinal pathogens (Platts-Mills et al., 2017). The TAC assay is regarded as a very sensitive molecular diagnostic technique for detecting a broad spectrum of enteropathogens. Moreover, by incorporating a wide range of socio-demographic variables, we were able to mitigate the influence of possible confounding factors. Furthermore, the study benefits from a large sample size and an extensive questionnaire, both of which are important aspects of the research.

Only limited work has been conducted in Bangladeshi adults about the relationship between *E. coli* pathotypes and EED biomarkers. Therefore, this study offers current information and highlights the importance of *E. coli* pathotypes as significant diarrheagenic bacteria that have a negative effect on the gastrointestinal health of undernourished adults.

In conclusion, the study results indicate that the presence of *E. coli* pathotypes, such as EPEC and STEC, in the human intestine affects gut health. Additionally, all of these pathotypes may potentially have a pathogenic role when other organisms are present. The ongoing research can aid in the investigation of the mechanisms underlying EED with common *E. coli* pathotypes and potentially facilitate the development of vaccines. These findings can be used as evidence by public health services to inform the planning and development of therapeutic intervention programs for adults.

## Data Availability

The original contributions presented in the study are included in the article/**Supplementary Material.** Further inquiries can be directed to the corresponding author.
